# Role of the Endogenous Cannabinoid System in Nicotine Addiction: Novel Insights

**DOI:** 10.3389/fpsyt.2015.00041

**Published:** 2015-03-25

**Authors:** Islam Hany Gamaleddin, Jose M. Trigo, Aliou B. Gueye, Alexander Zvonok, Alexandros Makriyannis, Steven R. Goldberg, Bernard Le Foll

**Affiliations:** ^1^Translational Addiction Research Laboratory, Campbell Family Mental Health Research Institute, Centre for Addiction and Mental Health, Toronto, ON, Canada; ^2^Directorate of Poison Control and Forensic Chemistry, Ministry of Health, Riyadh, Saudi Arabia; ^3^Center for Drug Discovery, Bouvé College of Health Sciences, Northeastern University, Boston, MA, USA; ^4^Preclinical Pharmacology Section, Behavioral Neuroscience Research Branch, Intramural Research Program, National Institute on Drug Abuse, National Institutes of Health, Department of Health and Human Services, Baltimore, MD, USA; ^5^Alcohol Research and Treatment Clinic, Addiction Medicine Services, Ambulatory Care and Structured Treatments, Centre for Addiction and Mental Health, Toronto, ON, Canada; ^6^Department of Family and Community Medicine, Institute of Medical Sciences, University of Toronto, Toronto, ON, Canada; ^7^Department of Psychiatry, Institute of Medical Sciences, University of Toronto, Toronto, ON, Canada; ^8^Department of Pharmacology and Toxicology, Institute of Medical Sciences, University of Toronto, Toronto, ON, Canada

**Keywords:** cannabinoid system, nicotine, addiction, endogenous cannabinoids

## Abstract

Several lines of evidence have shown that the endogenous cannabinoids are implicated in several neuropsychiatric diseases. Notably, preclinical and human clinical studies have shown a pivotal role of the cannabinoid system in nicotine addiction. The CB_1_ receptor inverse agonist/antagonist rimonabant (also known as SR141716) was effective to decrease nicotine-taking and nicotine-seeking in rodents, as well as the elevation of dopamine induced by nicotine in brain reward area. Rimonabant has been shown to improve the ability of smokers to quit smoking in randomized clinical trials. However, rimonabant was removed from the market due to increased risk of psychiatric side-effects observed in humans. Recently, other components of the endogenous cannabinoid system have been explored. Here, we present the recent advances on the understanding of the role of the different components of the cannabinoid system on nicotine’s effects. Those recent findings suggest possible alternative ways of modulating the cannabinoid system that could have implication for nicotine dependence treatment.

## Introduction

Among addictive substances, nicotine use is one of the most prevalent worldwide. The World Health Organization (WHO) estimates that nearly six million tobacco smokers will die every year as a consequence of their tobacco use (1). Therefore, tobacco smoking represents the largest preventable cause of death in the world. Nicotine exerts its effects on the main neurotransmitter systems, such as acetylcholine, dopamine, noradrenaline, serotonin, opioid, glutamate, and gamma-aminobutyric acid (GABA) systems (2–7). There is also mounting evidence supporting the existence of a significant role of the endocannabinoid system in mediating the reinforcing and other addiction-related effects of nicotine. The close overlap of cannabinoid and nicotinic acetylcholine receptors (nAChRs) in certain brain areas such as the midbrain, known to mediate the reinforcing properties of nicotine, but also the hippocampus and the amygdala that are involved in nicotine-associated memory certainly facilitates the interaction between both systems (8–10). There is also evidence of the existence of modulatory interactions between endocannabinoid and cholinergic signaling systems (11–13). Behavioral experiments have shown specific functional interactions between nicotine and the endocannabinoid system that could be mediated by brain structures involved in motivation (14).

The cannabinoid system includes the cannabinoid CB_1_ and CB_2_ receptors, endogenous cannabinoids, and the processes responsible for their biosynthesis, cellular uptake, and metabolism (15–18). Endocannabinoids are synthesized on-demand and can activate cannabinoid CB_1_ and/or CB_2_ receptors (19, 20). CB_1_ receptors are believed to be the main mediators of the psychoactive properties of delta-9-tetrahydrocannabinol (THC), which is the main psychoactive component of cannabis (21). CB_1_ receptors are among the most abundant G-protein-coupled receptors in the central nervous system (CNS) (22). Cannabinoids modify the synaptic efficacy of central neuronal circuits involved in reward and other processes by acting at CB_1_ receptors located pre-synaptically (23). Although CB_2_ receptor protein can be detected in the brainstem neurons using western blotting and immunohistochemistry (24), yet, levels of expression of brain CB_2_ receptors are much lower than those of CB_1_ receptors (25, 26). CB2 receptor mRNAs were detected in certain regions of the rat brain such as, the cerebellum, cortex, and brainstem using reverse transcription polymerase chain reaction (RT-PCR) (24). In contrast to the predominant pre-synaptic localization of CB_1_ receptors in the brain, immunoreactivity studies suggest a more likely post-synaptic localization of CB_2_ receptors (25, 26).

There are several endocannabinoids, of which the most studied have been arachidonoyl ethanolamide (anandamide; AEA) and 2-arachidonoylglycerol (2-AG). Anandamide synthesis is regulated through the conversion of a minor phosphoglyceride, *N*-arachidonyl phosphatidylethanolamine (*N*-arachPE), through two possible pathways. The first pathway involves phospholipase D (NAPE-PLD) (27) and the second involves two enzymes, alpha beta hydrolase (ABH4) and glycerophosphodiesterase (GDE1) (28). The exact processes regulating the involvement of these enzymes in the synthesis of anandamide have not been fully elucidated. Cessation of endocannabinoid signaling is hypothesized to happen through the transport inside the cell and the later degradation by specific enzymes. It is further hypothesized that anandamide and 2-AG actually share same the intracellular transport mechanism (17, 23). Anandamide and 2-AG seem to diffuse passively through lipid membranes due their lipophilic nature. Nevertheless, the diffusion process might be facilitated by a selective carrier system (29, 30). After anandamide’s uptake inside the cell, the enzyme fatty acid amide hydrolase (FAAH) degrades it into arachidonic acid and ethanolamine (31, 32). FAAH and CB_1_ receptors are widely distributed in the CNS and show partial overlap. However, FAAH is mainly available at the post-synaptic neurons whereas CB_1_ receptors are located at the pre-synaptic neurons (33, 34). On the other hand, 2-AG has its own distinct structure and different biosynthesis and degradation pathways. Moreover, 2-AG appears to be formed under conditions different from those required for the synthesis of anandamide and is modulated by different pharmacological mechanisms. 2-AG is synthesized in response to cellular activation from arachidonic acid-containing membrane phospholipids. The most important pathway for 2-AG synthesis is the phosphatidylinositol (PI)-phospholipase C (PLC)/DAG lipase [diglyceride lipase (DAGL)] pathway, which involves the hydrolysis of inositol phospholipids by PLC. The second pathway for producing 2-AG is through the sequential hydrolysis of PI (35). Although a large number of enzymes are involved in the hydrolysis of monoacyl glycerols, evidence has shown that MAG lipase [monoglyceride lipase (MAGL)] might play a fundamental role on 2-AG degradation. The remaining 2-AG seems to be hydrolyzed by ABHD6 and ABHD12 enzymes (36), although the information on this regard is limited. Interestingly, it has been demonstrated that anandamide enhances the metabolism and in turn attenuates 2-AG effects in the striatum (37). These findings suggest that anandamide and 2-AG might have different actions according to the different physiological or pathophysiological conditions under which they are synthesized (17). Endocannabinoids function as non-conventional neuromodulators whose functions include retrograde signaling and they mediate several types of synaptic plasticity (38). Once released by post-synaptic neurons, endocannabinoids will inhibit neurotransmitter release by pre-synaptic neurons. The respective inhibition of GABA or glutamate release by endocannabinoids mediate depolarization-induced suppression of “inhibition” (DSI) (39, 40) or the depolarization-induced suppression of “excitation” (DSE) (41), respectively. The occurrence of DSE and DSI in the mesocorticolimbic system (42–44) is relevant because of the importance of this system in addiction.

Emerging evidence has shown that the endogenous cannabinoid ligands are implicated in drug addiction processes (10, 45, 46). We will review the literature on nicotine only. Initial preclinical and human clinical studies suggested that the use of CB_1_ receptor inverse agonists/antagonists such as rimonabant (also known as SR141716) and AM251 might be effective for the treatment of nicotine addiction (47). In fact, rimonabant was able to improve smoking cessation rates in controlled trials (47). However, the use of rimonabant was associated with higher rates of anxiety and depression (48, 49), and consequently this medication was withdrawn from the market in 2008 (50). In this article, we will review the recent advances that have occurred in the last few years regarding our understanding of the different components of the cannabinoid system and how it possibly modulates nicotine addiction.

## Nicotine Dependence and Brain Reward Pathways

To better interpret how endocannabinoids modulate the reinforcing effects of nicotine, it is important to understand how nicotine interacts with the different brain reward pathways, particularly the mesolimbic dopaminergic pathway. The psychoactive effects of nicotine are believed to occur through its activation of the nAChRs. These receptors are located in a variety of brain areas and are not limited to the central cholinergic pathways. The nAchRs have been detected in high densities in the thalamus and caudate nucleus, moderate densities in the frontal and temporal parietal cortices, and in the cerebellum with low levels in white matter tracts (51). Exposure to nicotine in a chronic manner leads to desensitization (52) and up regulation (53) of α4β2* subtype of high-affinity nAChRs (54). This receptor subtype has been shown to play a major role in mediating the reinforcing and antinociceptive effects of nicotine. In the ventral tegmental area (VTA), nicotine binds to nAChRs located on nerve terminals of GABAergic and glutamate neurons projecting on the dopaminergic neurons, but also on nAChRs located directly on dopamine neurons (55). The dopamine neurons project to several brain regions implicated in reward including the nucleus accumbens (NAc). Nicotine administration ultimately stimulates the release of dopamine in the dorsal and ventral striatal terminals, notably the NAc (52). These findings have been validated using positron emission tomography (PET) approach (56, 57). Notably, a recent PET study using [^11^C]-(+)-PHNO PET tracer have shown that tobacco smoking produced elevation of dopamine in the limbic striatum and in extra-striatal area (the ventral pallidum) (57). Interestingly, this study identified that in smokers, dopamine release in the limbic striatum was associated with motivation to smoke, anticipation of pleasure from cigarettes, and relief of withdrawal symptoms. Furthermore, studies have shown that the lesion of the mesolimbic dopamine system (58) or administration of selective dopamine antagonists results in a significant decrease of nicotine self-administration in rats (59). On the other hand, several neurotransmitter systems have shown to play a vital role in nicotine dependence. Studies have shown that nicotine-induced dopamine release can be reduced significantly by atropine (muscarinic receptor antagonist), eticlopride (dopamine D1/2 receptor antagonist), and MK801 [*N*-methyl-d-aspartate (NMDA) antagonist] (60). It has been shown that smoking cigarettes enhances plasma levels of endogenous opioids (61, 62) and that nicotine stimulates the release of β-endorphins in neuronal cell cultures (63). Furthermore, nicotine-conditioned place preference (CPP) and nicotine-induced antinociception were significantly attenuated in μ-opioid knockout mice compared to wild-type mice (64). Additionally, naloxone has been shown to block nicotine CPP in mice (65).

## Endocannabinoid Modulation of Dopaminergic Neuronal Inputs

The dopaminergic system has long been hypothesized to play an essential role in the formulation of goal-directed behaviors of natural rewards and drugs of abuse including nicotine (58, 66, 67). Furthermore, the conditioned-reinforcing properties of drugs of abuse and their associated stimuli are also mediated through the dopaminergic system (68, 69). Dopamine is further involved in the development of behavioral sensitization that follows the repeated administration of drugs of abuse, as well as non-drug stimuli (70).

Several lines of evidence have demonstrated the significance of the dopaminergic system in cue associations. Using a discriminative stimulus and a conditioned stimulus as conditioning tasks associated with a food reward, Miller and colleagues, demonstrated an increase in neuronal firing in the VTA and substantia nigra (71). Similarly, in monkeys, phasic neuronal responses were recorded in response to conditioned stimuli in dopaminergic neurons (72).

The modulatory role of the endocannabinoid system on signaling in the mesolimbic dopamine reward system (73, 74), is believed to be substantiated by its abundant presence within the VTA (75, 76). The ability of endocannabinoids to act as retrograde neurotransmitters (44) allows them to attenuate the activity of external afferents (pre-synaptic neurons) (42) and allows dopamine neurons (post-synaptic neurons) to regulate their own function (43). This topic has been recently reviewed by Wang and Lupica (77) and will be not developed here. It appears that the main endocannabinoid regulating dopamine firing is 2-AG and it has been proposed that the burst firing activity pattern of dopamine neurons as well as the long-term plasticity effects induced by drugs of abuse are regulated by 2-AG (77, 78). The critical role of endocannabinoids mediating the ability of drugs of abuse, including nicotine, to stimulate reward pathway is shown by multiple pharmacological studies (14, 79, 80).

## Role of CB_1_ Receptors on Nicotine Addictive Properties

**Figure 1:**
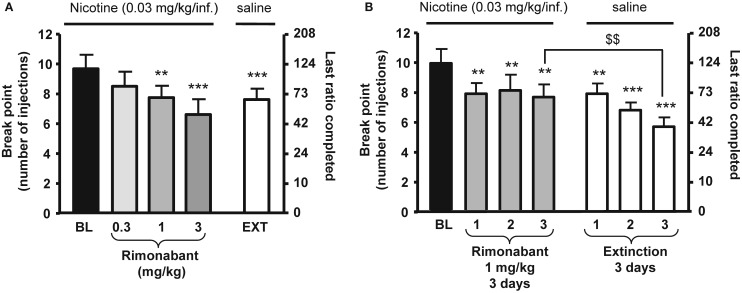
**Effects of rimonabant on motivation for nicotine in rats**. In **(A)**, rimonabant [0.3–3 mg/kg, IP 60 min pre-treatment time (PTT)] dose-dependently reduced nicotine (0.03 mg/kg/injection) self-administration under a progressive-ratio schedule. Data are expressed as means (±SEM) of the number of injections (break-point, left *y*-axis) and of the last ratio completed (in number of lever presses, right *y*-axis) during baseline (BL) conditions, rimonabant pre-treatment, and vehicle pre-treatment and substitution of nicotine with saline (EXT). *N* = 8. **p < 0.01; ***p < 0.001 vs. baseline (BL), Dunnett’s test after significant ANOVA for repeated measures. In **(B)**, effects of rimonabant (1 mg/kg, IP 60 min PTT) on nicotine self-administration under a progressive-ratio schedule during three consecutive sessions. Data are expressed as means (±SEM) of the number of injections (break-point, left *y*-axis) and of the last ratio completed (in number of lever presses, right *y*-axis) during baseline (BL) conditions, during three consecutive sessions with rimonabant pre-treatment (1 mg/kg) and during three consecutive sessions with vehicle pre-treatment and substitution of nicotine with saline. *N* = 9. **p < 0.01; ***p < 0.001 vs. baseline; ^$$^*p* < 0.01 vs. vehicle extinction group, Student Newman–Keuls multiple comparison test after significant ANOVA for repeated measures. The figure and its caption have been reproduced with permission from Ref. (81).

**Figure 2:**
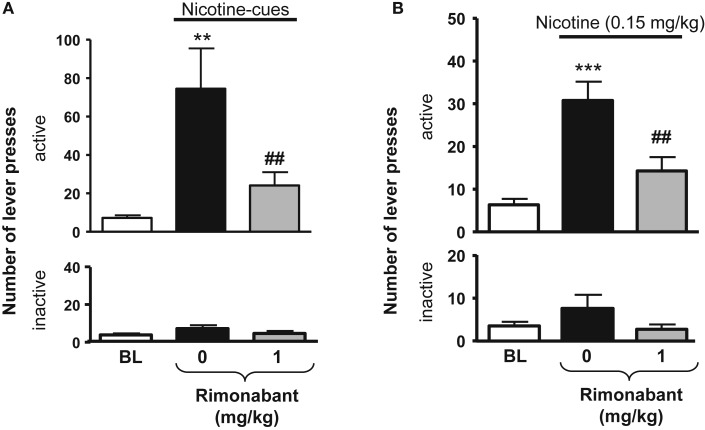
**Effects of rimonabant on nicotine-seeking in rats**. In **(A)**, effects of rimonabant [0.1 mg/kg, IP 60 min pre-treatment time (PTT)] on the active (top) and the inactive (below) levers responses during cue-induced reinstatement of nicotine-seeking. **p < 0.01 vs. baseline; ^##^*p* < 0.01 vs. vehicle pre-treatment. In **(B)**, effects of rimonabant (1 mg/kg, IP 70 min, PTT) on the active (top) and the inactive (below) levers responses during a nicotine-induced (0.15 mg/kg, SC, 10 min) reinstatement of nicotine-seeking. ***p < 0.001 vs. baseline; ^##^*p* < 0.001 vs. vehicle pre-treatment. The figure and its caption have been reproduced with permission from Ref. (81).

**Figure 3:**
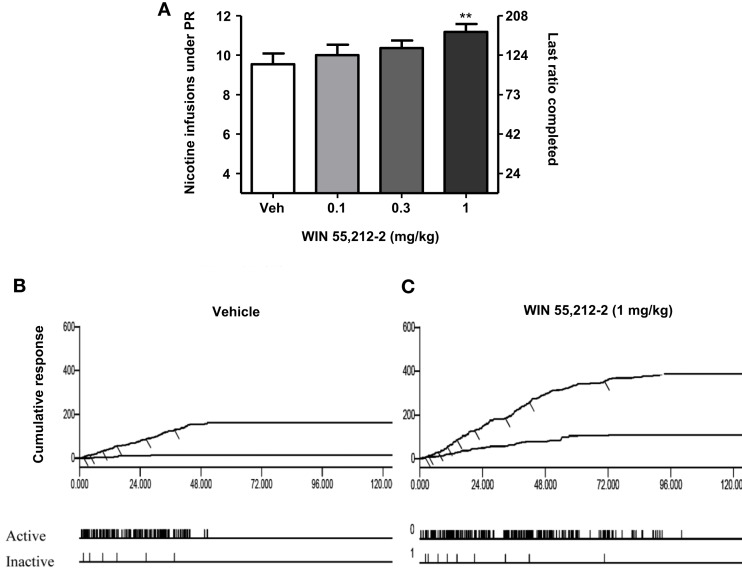
**Effects of the stimulation of cannabinoid receptors on motivation for nicotine**. In **(A)**, effects of pre-treatment with WIN 55,212-2 [0.1–1 mg/kg, IP 15 min pre-treatment time (PTT)] on nicotine (0.03 mg/kg/infusion) self-administration under a progressive-ratio schedule. Data are expressed as means (±SEM) of the number of infusions obtained during the 4-h sessions. **p < 0.01 vs. vehicle pre-treatment (Dunnett’s test after significant ANOVA for repeated measures *N* = 9). In **(B,C)**, individual representative cumulative responses on the active and inactive levers during nicotine self-administration under progressive-ratio schedule in rats pre-treated with vehicle **(B)** or 1 mg/kg WIN 55,212-2 **(C)**. Each short upward mark on the cumulative lever-press records indicates one nicotine infusion. Break-point values are indicated and the pattern of response across time on active and inactive levers is provided below. The figure and its caption have been reproduced with permission from Ref. (89).

**Figure 4:**
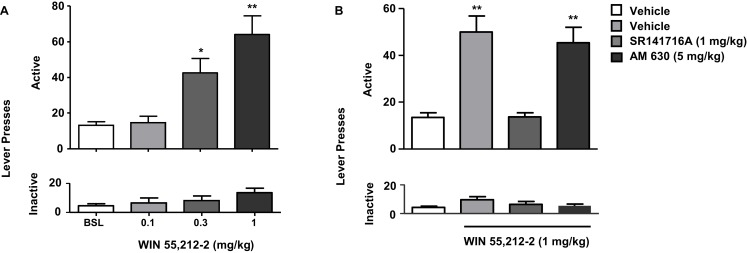
**Effects of the stimulation of cannabinoid receptors on nicotine-seeking**. In **(A)**, rats trained to self-administer nicotine underwent an extinction phase after which they were pre-treated with WIN 55,212-2 [0.1–1 mg/kg, IP 15 min pre-treatment time (PTT)]. Figure shows responses on the active lever (top) and inactive lever (bottom). WIN 55,212-2 (0.3 and 1 mg/kg) significantly reinstated nicotine-seeking, assessed by the number of responses on the active lever (*p < 0.05 and *p < 0.001). No significant changes in responding on the inactive lever were observed. In **(B)**, pre-treatment with the CB_1_ antagonist rimonabant (SR141716A) (1 mg/kg, IP), but not with the CB_2_ antagonist AM630 (5 mg/kg, IP) reversed reinstatement of nicotine-seeking induced by WIN 55,212-2. The figure and its caption have been reproduced with permission from Ref. (89).

In rats, the selective CB_1_ receptor inverse agonist/antagonist rimonabant decreases intravenous nicotine self-administration behavior and also nicotine-induced elevations in extracellular dopamine in the NAc (14). We and others subsequently reported that rimonabant decreases the motivation to self-administer nicotine, as measured using progressive-ratio schedules of reinforcement (81) (see Figure 1), blocks the development of nicotine-induced CPP (82–84), and the reinstatement of previously extinguished nicotine-seeking behavior in rats (81, 85, 86) (see Figure 2). Several studies have shown that genetic deletion of cannabinoid CB_1_ receptors reduces nicotine-induced CPP (87, 88). Cannabinoid CB_1_ receptor stimulation, in contrast, increased the motivation to self-administer nicotine as measured using a progressive-ratio schedule of reinforcement (89) (see Figure 3), enhanced cue-induced reinstatement of nicotine-seeking behavior and the discriminative stimulus effects of low doses of nicotine in rats (89) (see Figure 4). Several of these responses in rats were blocked by the CB_1_ inverse agonist/antagonist rimonabant. However, the CB_2_ antagonist AM630, was not able to block CB_1_ stimulation effects, supporting the critical role of CB_1_ receptors in mediating nicotine-dependent processes (89, 90). Interestingly, blockade of the CB_1_ receptors in the shell of the NAc, the basolateral amygdala, and the prelimbic cortex (91), but also in the bed nucleus of the stria terminalis (92), is able to reduce nicotine-seeking behavior. Nicotine-taking appears to be controlled by CB_1_ receptors located in the VTA, but not in the NAc (93). Taken together, these findings indicate that CB_1_ receptors have a bi-directional role on both nicotine reward/reinforcement and on relapse to nicotine-seeking behavior in abstinent subjects.

## CB_2_ Receptors on Nicotine Addictive Properties

Several lines of evidence indicate that central CB_2_ receptors could be implicated in modulating several neuropsychiatric disorders, including drug addiction (94–96). In fact, the effects of CB_1_ and CB_2_ receptor activation (at high doses) can produce similar effects such as antinociception and catalepsy (97, 98). However, the activation of CB_2_ receptors by the selective CB_2_ agonist AM1241 did not have effects on motivation to obtain nicotine or nicotine intake in rats (90). Similarly, the selective CB_2_ antagonist AM630 did not modify nicotine-taking or motivation to obtain nicotine under the progressive-ratio schedule of reinforcement. Moreover, the CB_2_ agonist and antagonist were not able to affect cue or nicotine-induced reinstatement of nicotine-seeking behavior (90). Together, these results further support the current literature on the distinct behavioral, neurochemical, and immunological profiles, of CB_1_ and CB_2_ receptors. On the other hand, it should be noted that in mice, CB_2_ receptors have been shown to modulate some addictive properties of drugs of abuse using both genetic and pharmacological models (99, 100). Another recent study by Zhang and colleagues, reported that activation of CB_2_ receptors in the VTA can attenuate cocaine self-administration in WT and CB_1_ knockout but not in CB_2_ knockout mice (101). Moreover, recent studies have documented the relevance of CB_2_ receptors on the rewarding/reinforcing properties of nicotine in mice (102, 103). Altogether, these results suggest that there may be important species differences that mediate these effects. Further studies performed in non-human primates or human subjects would allow better exploration of these discrepancies.

## Role of Endogenous Cannabinoid Ligands in Modulating the Reinforcing Effects of Nicotine

A recent study have shown that the volitional intake of nicotine (i.e., nicotine self-administration in rats) was able to modify anandamide and oleoylethanolamide (OEA) levels in the VTA (104). This is an interesting finding considering that several studies have described the existence of modulatory effects in signaling between the endocannabinoid and cholinergic systems (11–13). The understanding of effects of anandamide and 2-AG in modulating nicotine-reinforcing properties has been facilitated by the discovery of drugs able to interfere with the different processes involved in the synthesis, reuptake, and inactivation of these endocannabinoids. Among those drugs, *N*-(4-hydroxyphenyl)-arachidonamide (AM404) and cyclohexyl carbamic acid 3′-carbamoyl-3-yl ester (URB597) produce elevation of anandamide levels by blocking anandamide reuptake or by inhibiting FAAH, respectively (105, 106).

Based on observations of the effects of cannabinoid receptor agonists and antagonists on nicotine’s rewarding properties, one could speculate that increasing brain anandamide levels might enhance nicotine’s rewarding/reinforcing effects. Consistent with this prediction, Merritt et al. (88) observed enhanced nicotine CPP in FAAH knockout mice. Similarly, the pre-treatment with URB597 enhanced nicotine CPP in mice (88). Administration of a sub-threshold dose of nicotine that did not produce CPP in wild-type mice effectively produced CPP in FAAH knockout mice, and this effect was mediated by CB_1_ receptors (88). The enhancement of nicotine’s rewarding effects in FAAH knockout mice further supports previous studies where co-administration of sub-threshold doses of nicotine and THC produced nicotine CPP in mice (107). On the other hand, no differences on nicotine CPP were observed between FAAH knockout and wild-type mice when higher doses of nicotine were tested (88).

In a marked contrast to the results of Merritt et al. (88) in mice, FAAH inhibition by URB597 has been shown to reverse some addiction-related behavioral and neurochemical effects of nicotine in rats (81, 108). The inhibition of FAAH by URB597 was able to prevent the development of nicotine-induced CPP, reduced acquisition of nicotine self-administration behavior, and inhibited reinstatement of nicotine-seeking behavior induced by nicotine priming and cue-induced reinstatement in abstinent rats, while demonstrating no rewarding effects *per se* (108). However, there was no impact on nicotine-taking assessed using a fixed-ratio or a progressive-ratio schedule of reinforcement (81). A possible explanation for the differences observed between those studies is the fact that different species were used in these studies: rats (81, 108) vs. mice (88). However, URB597-induced increases in anandamide brain levels do not differ between mice and rats (109).

## Possible Involvement of Non-Cannabinoid Systems FAAH Inhibitors Effects

There is a surprising similarity between the effects of FAAH inhibition by URB597, which is expected to enhance anandamide levels and, thus, enhance cannabinoid CB_1_ receptor signaling, and those of rimonabant, described earlier, which blocks cannabinoid CB_1_ receptor signaling. In addition to a similarity in behavioral effects, it has been shown that both compounds, URB597 and rimonabant, were able to block nicotine-induced increases of dopamine levels in the NAc (14, 108). The similar effects of URB597 and rimonabant described above could be explained by the involvement of non-cannabinoid peroxisome proliferator-activated nuclear receptor (PPAR-α) systems (110, 111). Therefore, PPAR-α receptors seems to mediate the effects of FAAH inhibition on nicotine’s abuse-related behavioral and neurochemical effects in both rats and monkeys while CB_1_ receptors may play the major role in mediating the effects of FAAH inhibition on nicotine’s abuse-related behavioral and neurochemical effects in mice. This is supported by findings by Fegley et al. (109), showing that 2 h after treatment with 0.3 mg/kg URB597, there was only a twofold increase in brain levels of the endogenous PPAR-α ligand OEA and palmitoylethanolamide (PEA) in mice compared to a four to fivefold increase in OEA and PEA levels in rats (109). In contrast, as commented above, URB597-induced increases in anandamide brain levels do not differ between mice and rats (109). Moreover, the PPAR-α receptor antagonist MK-886 blocked URB597-induced reductions in nicotine’s effects on dopaminergic neuronal activity in rats (112). Similar to URB597, a variety of natural and synthetic PPAR-α receptor agonists were shown to decrease nicotine-reinforcing properties and reinstatement nicotine-seeking in different species (111). Another mechanism by which PPAR-α ligands are proposed to modulate the reinforcing effects of nicotine is downstream that activation of α7-nAChR subtype. During low activity, acetylcholine preferentially binds to high-affinity β2-nAChRs. This binding does not trigger nAChRs-mediated modulation of PPAR-α ligands. However, upon activation of cholinergic receptors, low affinity α7-nAChRs located in the extra dendritic regions of dopaminergic neurons are activated. This activation leads to an increase in intracellular Ca2+ which stimulates the synthesis of the PPAR-α ligands OEA and PEA as well as, anandamide. These ligands in turn activate PPAR-α which exerts negative modulation of β2-nAChRs through tyrosine kinase-mediated phosphorylation of β2-nAChRs. This mechanism demonstrates how dopaminergic neurons in the VTA have an ability to self-regulate their firing through selectively increasing OEA and PEA levels (110, 113). Thus, PPAR-α receptors could also be mediating their inhibitory effect on nicotine’s addiction-related behavioral and neurochemical effects through a non-FAAH pathway (111).

Anandamide might also modulate nicotine effects by targeting other receptors such as transient potential receptor of vanilloid type 1 (TRPV1) or even nicotine receptors. Indeed, anandamide has been shown to inhibit α4β2-nAChRs function in a CB_1_ receptor-independent manner (114, 115). Therefore, the effect of endocannabinoids on nicotine-reinforcing properties seems to be complex and suitable of being affected by different variables including the species and nicotine doses being tested. Other non-cannabinoid systems as vanilloid and PPAR-α might also be involved.

## Similarities between FAAH Inhibitors and Endocannabinoid Uptake Inhibitors Effects

**Figure 5:**
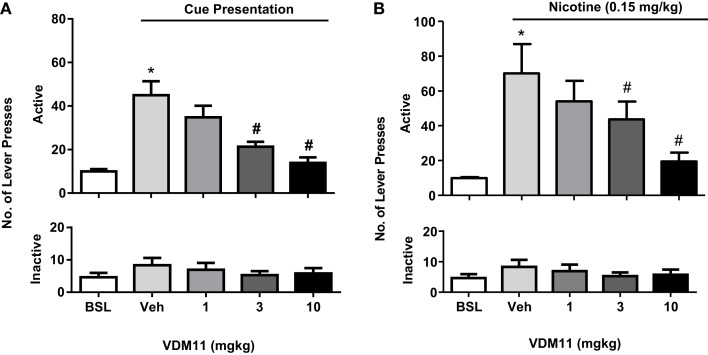
**Effects of VDM11 on cue and nicotine priming reinstatement of nicotine-seeking behavior**. In **(A)**, a significant reinstatement of nicotine- seeking behavior was produced by presentation of nicotine-associated cues in non-treated animals (*p < 0.01). Pre-treatment with VDM11 [3 and 10 mg/kg, IP 30 min pre-treatment time (PTT)] significantly reduced cue-induced reinstatement of nicotine-seeking behavior (^#^*p* < 0.05). In **(B)**, a significant reinstatement of nicotine-seeking was also produced by pre-treatment with nicotine (0.15 mg/kg) (*p < 0.01). VDM11 (3 and 10 mg/kg, IP 30 min PTT) significantly reduced the reinstatement of nicotine-seeking behavior induced by a priming injection of 0.15 mg/kg nicotine administered 10 min before the session (^#^*p* < 0.05). Data are expressed as means (±SEM) of the number of active lever presses during extinction (BSL) vehicle pre-treatment (visual cues). The figure and its caption have been reproduced with permission from Ref. (116).

In support of the results obtained with the FAAH inhibitor URB 597, the anandamide uptake inhibitor VDM11 (5Z, 8Z, 11Z, 14Z)-*N*-(4-hydroxy-2-methylphenyl)-5, 8, 11, 14-eicosatetraenamide, was able to reduce both cue- and nicotine-induced reinstatement of nicotine-seeking behavior. However, VDM11 did not affect responding for nicotine under fixed-ratio or progressive-ratio schedules of reinforcement (116) (see Figure 5). Findings with VDM11 were further confirmed using AM404, another anandamide uptake inhibitor, which also attenuated cue and nicotine priming reinstatement of nicotine-seeking behavior (117). Additionally, AM404 decreased nicotine CPP and its reinstatement. AM404 has been also shown to attenuate dopamine increase on the NAc shell following nicotine injection (118). The fact that two different ligands developed to elevate anandamide levels produce similar effects, strongly suggest that those effects are mediated by anandamide elevation.

The effects observed with VDM11 and AM404 on nicotine reward/reinforcement, were further supported by Oleson and Cheer (119) using the intracranial brain stimulation paradigm. Elevating levels of anandamide using the anandamide uptake inhibitor VDM11 reduced NAc neural encoding of reward-predictive cues and attenuated reward seeking, defined as the time occurring between cue presentation and a reward-directed behavioral response (120). A possible explanation for these findings is that VDM11 increases anandamide to a greater extent than 2-AG *in vivo* (121). Since anandamide functions as a partial agonist, the elevation of its levels in the brain induced by VDM11 might allow it to compete with 2-AG, a full CB_1_ receptor agonist (122), thereby anandamide might block 2-AG effects on reward seeking. In fact, comparing different behavioral studies suggest that anandamide and 2-AG may have opposite effects on reward-seeking behavior (81, 116, 119, 120). Interestingly, anandamide and 2-AG are effective reinforcers as evaluated using the intravenous drug self-administration paradigm in squirrel monkeys (123, 124). However, while 2-AG increases dopamine neurotransmission (125) and facilitates reward-directed behavior (125, 126), the elevation of anandamide levels attenuates the ability of cues to motivate reward-seeking behavior (81, 116, 119, 120).

It has been suggested that VDM11 might be actually a substrate for FAAH. Thus, it has been shown that VDM11 reduces FAAH hydrolysis of anandamide *in vitro* [(127, 128), also see Ref. (129)]. Therefore, the above described effects of VDM11 attenuating reward seeking might be due FAAH inhibition as well as reduced anandamide uptake. In that case, other fatty acid amides affected by FAAH catabolic effects, such as PEA and OEA, might also potentiate the effects of anandamide at TRPV1 receptors (130). As the specific pharmacological mechanisms of action of endocannabinoids remain unclear, it would be interesting to investigate the brain regions involved in mediating the effects observed with increasing levels of anandamide (e.g., intraregional injection of anandamide uptake inhibitors in areas of the brain that may be involved in mediating reward/reinforcement such as the basolateral amygdala and the NAc shell) (131). The ubiquitous nature of the endocannabinoid system might provide another interpretation regarding the pharmacological mechanism of action by which anandamide uptake inhibitors modulate neural mechanisms of reward seeking (132–134).

An alternative explanation of how VDM11 might attenuate reward seeking comes from the pharmacological inhibition of the membrane transporter. The bi-directional role of the membrane transporter in transporting anandamide and 2-AG (29, 30, 135) might explain the effects of VDM11 in reward seeking. In fact, recent studies using VDM11 have reported effects that are in close resemblance to those observed following CB_1_ receptor blockade (116, 119).

## Endocannabinoids and Nicotine Withdrawal

Endocannabinoids seem to be implicated in the response to withdrawal from nicotine. The concomitant treatment with nicotine and THC in mice induced an increased withdrawal syndrome when these mice were challenged with rimonabant (107). Additionally, the administration of THC seems to attenuate the magnitude of nicotine withdrawal (136). On the other hand, rimonabant failed to induce withdrawal in nicotine-dependent animals (136), suggesting that the cannabinoid system is not involved in the expression of nicotine physical dependence. However, more recent findings have reported modification in the number of nicotine receptors following chronic exposure to cannabinoids (137). Additionally, Cippitteli and colleagues reported that withdrawal from nicotine is associated with fluctuations in anandamide but not in 2-AG (138). Interestingly, the same study showed that administration of URB597, decreased nicotine withdrawal associated anxiety, but did not alter somatic signs of nicotine withdrawal (138).

## Conclusion

It appears, from the experiments conducted in preclinical models (and partly validated by the testing of rimonabant in human clinical trials) that the CB_1_ receptors are critically involved in mediating nicotine reward/reinforcement. CB_1_ receptors also seem to be involved in mediating cue and nicotine priming reinstatement of nicotine-seeking behavior. Further work is needed to explore the role of CB_1_ receptors in nicotine-seeking induced by stress. It appears that many of these findings hold true in non-human primate studies as well as rodents (46, 47, 139). Thus, the CB_1_ receptor appears to be a logical target for drug development. As inverse agonist/antagonists have been found to have negative side-effects in humans (i.e., anxiety/depression), novel approaches, such as neutral CB_1_ receptor antagonists that might be effective against drug addiction without inverse agonist/antagonists side-effects, could represent a better therapeutic option (140–142). Limited experiments conducted in rats, suggest that the CB_2_ receptor has no involvement in mediating nicotine self-administration and relapse to nicotine-seeking. However, since differences have been reported in the role of CB_2_ receptors in mice and rats, it would be interesting to further explore the role of CB_2_ receptors before concluding that this receptor is not an interesting target. Recent experiments suggest that elevating brain anandamide levels would be an effective strategy to reduce relapse to nicotine-seeking behavior, but not to reduce ongoing nicotine-taking behavior. This is of great interest as such a strategy has been shown to decrease anxiety and depression in some animal models and may therefore be better tolerated than CB_1_ receptor inverse agonist/antagonists. However, we have no validation in humans of these interesting findings. Finally, exploring the role of the endogenous cannabinoid receptor ligand 2-AG on nicotine-taking and -seeking behavior will improve our understanding of the respective behavioral role of different endocannabinoids. Overall, those recent studies suggest that the endocannabinoid system still presents many opportunities for development of novel therapeutic strategies for nicotine addiction.

## Conflict of Interest Statement

The authors declare that the research was conducted in the absence of any commercial or financial relationships that could be construed as a potential conflict of interest.
